# Neurovascular coupling and cerebrovascular hemodynamics are modified by exercise training status at different stages of maturation during youth

**DOI:** 10.1152/ajpheart.00302.2023

**Published:** 2023-07-14

**Authors:** Jack S. Talbot, Dean R. Perkins, Tony G. Dawkins, Andrew J. M. Douglas, Thomas D. Griffiths, Cory T. Richards, Kerry Owen, Rachel N. Lord, Christopher J. A. Pugh, Jon L. Oliver, Rhodri S. Lloyd, Philip N. Ainslie, Ali M. McManus, Mike Stembridge

**Affiliations:** ^1^Cardiff School of Sport and Health Sciences, Cardiff Metropolitan University, Cardiff, United Kingdom; ^2^Centre for Health, Activity and Wellbeing Research, Cardiff Metropolitan University, Cardiff, United Kingdom; ^3^Department of Sport Science, University of Innsbruck, Innsbruck, Austria; ^4^Centre for Heart, Lung and Vascular Health, School of Health and Exercise Sciences, University of British Columbia Okanagan, Kelowna, British Columbia, Canada; ^5^Windsor Clive Primary School, Cardiff, United Kingdom; ^6^Youth Physical Development Centre, Cardiff Metropolitan University, Cardiff, United Kingdom; ^7^Sports Performance Research Institute New Zealand, AUT University, Auckland, New Zealand; ^8^Centre for Sport Science and Human Performance, Waikato Institute of Technology, Waikato, New Zealand

**Keywords:** adolescence, exercise, neurovascular coupling

## Abstract

Neurovascular coupling (NVC) is mediated via nitric oxide signaling, which is independently influenced by sex hormones and exercise training. Whether exercise training differentially modifies NVC pre- versus postpuberty, where levels of circulating sex hormones will differ greatly within and between sexes, remains to be determined. Therefore, we investigated the influence of exercise training status on resting intracranial hemodynamics and NVC at different stages of maturation. Posterior and middle cerebral artery velocities (PCA_v_ and MCA_v_) and pulsatility index (PCA_PI_ and MCA_PI_) were assessed via transcranial Doppler ultrasound at rest and during visual NVC stimuli. *N* = 121 exercise-trained (males, *n* = 32; females, *n* = 32) and untrained (males, *n* = 28; females, *n* = 29) participants were characterized as pre (males, *n* = 33; females, *n* = 29)- or post (males, *n* = 27; females, *n* = 32)-peak height velocity (PHV). Exercise-trained youth demonstrated higher resting MCA_v_ (*P* = 0.010). Maturity and training status did not affect the ΔPCA_v_ and ΔMCA_v_ during NVC. However, pre-PHV untrained males (19.4 ± 13.5 vs. 6.8 ± 6.0%; *P* ≤ 0.001) and females (19.3 ± 10.8 vs. 6.4 ± 7.1%; *P* ≤ 0.001) had a higher ΔPCA_PI_ during NVC than post-PHV untrained counterparts, whereas the ΔPCA_PI_ was similar in pre- and post-PHV trained youth. Pre-PHV untrained males (19.4 ± 13.5 vs. 7.9 ± 6.0%; *P* ≤ 0.001) and females (19.3 ± 10.8 vs. 11.1 ± 7.3%; *P* = 0.016) also had a larger ΔPCA_PI_ than their pre-PHV trained counterparts during NVC, but the ΔPCA_PI_ was similar in trained and untrained post-PHV youth. Collectively, our data indicate that exercise training elevates regional cerebral blood velocities during youth, but training-mediated adaptations in NVC are only attainable during early stages of adolescence. Therefore, childhood provides a unique opportunity for exercise-mediated adaptations in NVC.

**NEW & NOTEWORTHY** We report that the change in cerebral blood velocity during a neurovascular coupling task (NVC) is similar in pre- and postpubertal youth, regardless of exercise-training status. However, prepubertal untrained youth demonstrated a greater increase in cerebral blood pulsatility during the NVC task when compared with their trained counterparts. Our findings highlight that childhood represents a unique opportunity for exercise-mediated adaptations in cerebrovascular hemodynamics during NVC, which may confer long-term benefits in cerebrovascular function.

## INTRODUCTION

The matching of cerebral perfusion to fluctuations in neural activity, termed neurovascular coupling (NVC), is essential for supporting cerebral metabolism and neurocognitive health (1). Childhood and adolescence represent critical periods of neurocognitive, neurovascular, and cardiovascular development (2–6). Accordingly, optimizing NVC during childhood and adolescence benefits long-term cardiovascular, neurocognitive, and mental health (7–9), but it is unclear what factors modify cerebral perfusion for a given cognitive task during adolescence (10, 11). When expressed as the change in cerebral perfusion during spontaneous fluctuations in resting neuronal activity, NVC declines across adolescence (10). The natural decline may be underpinned by axon myelination and synaptic pruning that aim to make neural connections more efficient (3, 6), so that the same neural impulse will place a lower metabolic demand in an adolescent teenager when compared with a child. However, it is not clear whether the NVC response to a cognitive task, rather than just during the resting state, is modified during pubertal maturation, partly due to the difficulty in selecting an age-appropriate test across the adolescent spectrum (11).

Adolescence is a period of youth characterized by marked, but sex specific, changes in insulin-like growth factor 1 (IGF-1) and sex hormones, which are known to mediate cerebral angiogenesis, endothelial function, and cerebrovascular tone during physiological stimuli in adults (12–16). Indeed, the developmental trajectory of resting cerebral perfusion is modified by biological sex across puberty (5, 12). Furthermore, reductions in nitric oxide availability and IGF-1 in older adults contribute to the progression of neurocognitive disease via cerebrovascular endothelial dysfunction (17, 18). Estrogen acts to increase neuronal excitability via glutaminergic neurotransmissions (15), while also improving endothelial function via increased expression of endothelial nitric oxide (NO) synthase (16). On the other hand, in males, influxes in androgens can impair endothelial function (17) by reducing NO availability (18). Thus, one may expect a sex-specific change in NVC across the pubertal period.

The process of long-term cerebrovascular and neurocognitive decline may begin early in life, as the combination of low cardiorespiratory fitness (V̇o_2max_) and low cognitive performance at 18 years of age has been associated with a hyperadditive risk of neurocognitive disease in males with advancing age (7). Endurance exercise training has consistently been shown to increase NO availability (19–21) and promote cerebral angiogenesis (14, 22). Therefore, critical periods of neural development during adolescence may represent a window of opportunity for external stimuli such as exercise to further stimulate the development of NVC.

Despite clear mechanistic evidence demonstrating the role of maturation and endurance training in modifying cerebrovascular function, it remains to be determined whether exercise training across the adolescent spectrum modifies NVC. Therefore, the aim of this study was to investigate the impact of exercise training status on intracranial hemodynamics and the NVC response in males and females at different stages of adolescence. We hypothesized that *1*) post-PHV endurance-trained youth would demonstrate elevated intracranial blood velocities compared with their untrained counterparts, whereas there would be no training-related differences in pre-PHV participants; and *2*) pre-PHV and post-PHV endurance-trained youth would demonstrate a larger NVC response than untrained counterparts during the NVC task.

## MATERIALS AND METHODS

### Ethical Approval

Ethical approval was granted by Cardiff Metropolitan University’s School of Sport and Health Sciences Research Ethics Committee (PGR-1339), and the study conformed to the Declaration of Helsinki (2013), except for registration in a database. Detailed, age-appropriate summaries of the methods and study design were given verbally and in writing to each participant before providing written assent. Furthermore, a legal guardian of each participant was given a verbal and written explanation of the methods and study design before providing written informed consent.

### Experimental Design

A group of youths (*n* = 163) volunteered to participate in the study. Participants were excluded if they failed to attend all laboratory visits (*n* = 3) or failed to meet our cohort health or physical activity criterion (*n* = 6). Based on self- and parental-reported physical activity, *n* = 154 participants were categorized as either endurance-trained (males, *n* = 42, age = 9.0–17.1 yr; females, *n* = 45, age = 8.2–17.0 yr) or untrained (males, *n* = 31, age = 8.0–17.7 yr; females, *n* = 36, age = 8.0–17.6 yr). “Trained” youths had completed ≥ 3 structured endurance exercise training sessions per week for ≥12 mo and were recruited from local-endurance sport clubs (see Table 1 for training volume data). “Untrained” youths were recruited from local schools and community clubs and were not taking part in regular endurance exercise training or meeting UK Chief Medical Officers’ Physical Activity Guidelines for children and young people (23). Following eligibility screening, participants attended the laboratory at Cardiff Metropolitan University on one occasion. Per technical guidelines for the assessment of cerebral blood flow (24), participants refrained from vigorous exercise, caffeine, and alcohol for ≥12 h before the data collection. Similar to comparable pediatric studies, participants attended the laboratory having fasted for ≥4 h (25, 26).

**Table 1.** Anthropometric and training status-related characteristics of participants

	Pre-PHV				Post-PHV				*P* Value (Interaction)						
	Untrained males	Trained males	Untrained females	Trained females	Untrained males	Trained males	Untrained females	Trained females	Maturation status	Sex	Training status	Maturation × sex	Maturation × training	Sex × training	Maturation × sex × training
*n*	15	18	13	16	13	14	16	17							
Maturation offset, yr	−2.7 (1.1)†	−2.4 (1.1)	−1.9 (0.7)	−1.6 (0.9)†	2.2 (0.8)*	1.8 (0.9)*	1.6 (0.9)*	2.1 (1.1)*	**≤0.001**	0.127	0.430	**0.013**	0.531	0.356	0.148
Age, yr	10.6 (1.6)	11.2 (1.7)	9.9 (1.2)	10.1 (1.2)	16.4 (1.0)	15.5 (0.9)	13.8 (1.6)	14.3 (1.4)	**≤0.001**	**≤0.001**	0.496	0.067	0.130	0.528	0.055
Weight, kg	38.9 (9.6)	36.2 (7.8)	33.7 (6.1)	3.25 (6.1)	65.0 (9.7)	62.7 (9.9)	51.3 (8.1)	55.4 (8.3)	**≤0.001**	**≤0.001**	0.728	0.052	0.342	0.187	0.427
Height, cm	145.7 (10.4)	145.5 (9.9)	137.7 (7.1)†	139.8 (8.8)†	179.3 (8.2)*	176.5 (8.9)*	161.1 (5.3)*†	165.4 (6.9)*†	**≤0.001**	**≤0.001**	0.570	**0.012**	0.955	0.129	0.437
Lean body mass, kg	29.8 (5.2)	31.0 (6.1)	25.5 (3.0)†	26.6 (4.6)†	53.5 (5.5)*	54.6 (6.9)*	39.3 (5.4)*†	44.3 (6.3)*†	**≤0.001**	**≤0.001**	**0.040**	**≤0.001**	0.366	0.360	0.318
MAP, mmHg	76 (6)	74 (5)	76 (5)	75 (7)	81 (7)	81 (5)	78 (5)	80 (6)	**≤0.001**	0.33	0.845	0.239	0.319	0.347	0.920
Training volume, h/wk	1.2 (0.9)	6.9 (2.3)‡	1.0 (1.0)	6.2 (2.0)‡	0.8 (0.8)	10.8 (2.9)*‡	0.6 (0.9)	9.3 (3.0)*‡	**≤0.001**	0.104	**≤0.001**	0.636	**≤0.001**	0.231	0.712
V̇o_2max_, mL·min·kg LBM^0.93^	46.7 (7.2)	54.3 (6.3)‡	44.2 (4.3)	49.3 (5.0)†‡	45.5 (5.7)	56.1 (5.0)‡	40.3 (4.0)†	50.5 (3.9)†‡	0.580	**≤0.001**	**≤0.001**	0.428	**0.044**	0.496	0.600

Values are means (SD); *n*, number of participants. LBM, lean body mass; MAP, mean arterial blood pressure; PHV, peak height velocity; V̇o_2max_, maximal oxygen consumption. Group differences were assessed via three-way ANOVA. *Significant difference between pre- and post-PHV youth. †Significant difference between males and females. ‡Significant difference between trained and untrained youth. Boldface indicates significant values at *P* ≤ 0.05.

Data collection was conducted in a quiet, temperature-controlled room with great care to minimize any external sensory stimulation during cerebrovascular measures. Upon arrival, participants completed a series of questionnaires quantifying their weekly exercise training or physical activity levels which were corroborated with parents, before completing anthropometric measurements. Participants were then instructed to lie down in the supine position, where they were fitted with instrumentation for the acquisition of cerebrovascular and cardiorespiratory data. Following baseline measures, we assessed NVC during a visual searching task previously used in pediatric cohorts (27). Thirty minutes after the NVC assessment, cardiorespiratory fitness (V̇o_2max_) was then determined via an incremental exercise test to volitional exhaustion and confirmed via a supramaximal verification of V̇o_2max_ on the same cycle ergometer as recommended for pediatric exercise testing (28, 29).

### Anthropometrics and Estimated Maturity Status

Body mass (kg) was measured using electronic scales, and stature (cm) and sitting height (cm) using a stadiometer, with participants barefoot and wearing light clothing. Anthropometrics, chronological age, and sex were entered into sex-specific regression equations to calculate maturity offset (predicted age from PHV), an estimate of somatic maturation (30). Participants were classified into pre- and post-PHV groups using ≥0.5 yr before and post-PHV, respectively, to account for the reported measurement error in the prediction equation. Skin fold thickness (skin fold callipers, Harpenden, Baty International, Burgess Hill, West Sussex, UK) was assessed at the triceps and subscapular for the estimation of lean body mass (LBM) as previously described (31, 32).

### Resting Cerebrovascular and Cardiorespiratory Measures

Resting measurements were acquired following ≥15 min of supine rest. During this time, participants wore a bilateral headset with 2-MHz TCD ultrasound probes (Spencer Technologies, Seattle, WA) placed over the temporal acoustic windows and adjusted to obtain MCA_v_ and PCA_v_. The M-1 segment of MCA was insonated on the right side of the head, whereas the P-1 segment of the PCA was insonated on the left side. MCA_v_ and PCA_v_ were identified and optimized according to their signal depth and waveform, and subsequently confirmed with a visual stimulation test as per the recommended TCD technique guidelines (33). The 2-MHz TCD ultrasound probes were secured and fastened in place once suitable MCA_v_ and PCA_v_ signals were confirmed.

All cardiorespiratory variables were sampled continuously at 1 kHz via an analog-to-digital converter (Powerlab 16/30, AD Instruments, Oxford, UK). Mean arterial blood pressure (MAP) and heart rate (HR) were measured by finger photoplethysmography (Finometer PRO, Finapres Medical Systems, Amsterdam, The Netherlands). Both $\text{P}{\text{ET}}_{{\text{CO}}_{2}}$ and $\text{P}{\text{ET}}_{{\text{O}}_{2}}$ and Pet_O_2__ were sampled via insertion of a sample line into a mouthpiece worn by the participant that connected in series to a bacteriological filter and a calibrated gas analyzer (ML206, AD Instruments Ltd, Oxford, UK). All data were interfaced with LabChart (v. 8) for subsequent off-line data analysis.

Cerebral blood velocities were also used to calculate the pulsatility index of the MCA (MCA_PI_) and PCA (PCA_PI_) at rest and during the NVC stimulus. Cerebral blood pulsatility index provides an index of downstream cerebrovascular resistance, which increases during older adulthood and may be related to a decline in cerebral metabolism and cerebrovascular dysfunction (34–36). Pulsatility index increases when the relative contribution of the peak systolic portion of the waveform to mean blood velocity increases, or when the contribution of the end-diastolic portion of the waveform decreases. As such, MCA_PI_ and PCA_PI_ were calculated as follows:

(Peak systolic velocity − minimum diastolic velocity)/mean velocity

Furthermore, cerebrovascular conductance of the MCA (MCA_CVC_) and PCA (PCA_CVC_) was calculated to account for the expected maturity-related differences in MAP across pre- and post-PHV youth (37), and to ensure group differences in intracranial velocities were not due to differences in cerebral perfusion pressure (38). Both MCA_CVC_ and PCA_CVC_ were calculated as follows:

Mean velocity/mean arterial blood pressure

### Neurovascular Coupling

Following resting measurements, participants remained in the supine position and a visual task was used to activate the visual cortex. Bilateral TCD was used to insonate the left PCA and right MCA through the transtemporal windows, allowing for selective increases in PCA_v_, and a regional comparison via the MCA_v_. Baseline PCA_v_, MCA_v_, HR, MAP, $\text{P}{\text{ET}}_{{\text{CO}}_{2}}$, and $\text{P}{\text{ET}}_{{\text{O}}_{2}}$ were acquired during a 2-min eyes-open and a 2-min eyes-closed trial before the visual stimulus. A portable device (iPad Air, Apple Distribution International, Hollyhill, Republic of Ireland) with a 19 cm × 15 cm visual field was then held 30–35 cm directly above the participant’s face throughout the NVC assessment. PCA_v_, MCA_v_ HR, blood pressure, $\text{P}{\text{ET}}_{{\text{CO}}_{2}}$, and $\text{P}{\text{ET}}_{{\text{O}}_{2}}$ were recorded continuously across five cycles of 30 s of eyes closed and 30 s of eyes open, consistent with other NVC research (39, 40).

The visual stimulus used in this experiment required the participant to search for an on-screen object (“Waldo”) that was hidden in a field of distractors consisting of similarly shaped characters in a variety of colors (41). The visual stimulus was chosen because of its previous use in assessing NVC in adults (41–43) and its use in vision research in youth (27). Furthermore, this visual stimulus may be particularly useful for the assessment of NVC in pediatric cohorts due to its better NVC signal-to-noise ratio compared with other paradigms (41).

### Data Processing

The interpolated PCA_v_, MCA_v_, HR, MAP, $\text{P}{\text{ET}}_{{\text{CO}}_{2}}$, and $\text{P}{\text{ET}}_{{\text{O}}_{2}}$ signals were visually inspected for artefacts or noise and corrected by cubic spline interpolation and downsampled to 10 Hz. Acceptable PCA_v_ and MCA_v_ waveforms were exported on a breath-by-breath and beat-by-beat basis and time aligned to HR, blood pressure, $\text{P}{\text{ET}}_{{\text{CO}}_{2}}$, and $\text{P}{\text{ET}}_{{\text{O}}_{2}}$ data, which were cubic spline interpolated at 5 Hz using a custom-built MATLAB code (The MathWorks, Natick, MA) (44). Data from each trial were aligned to stimulus onset (eyes open), and then averaged to generate one response per participant. The percent change in PCA_v_ and MCA_v_, as well as PCA_PI_ and MCA_PI_, was calculated from the average of the 5-s preceding initiation (i.e., the last 5 s of “eyes closed”) to the peak response during the subsequent “eyes open” to control for group differences in baseline PCA_v_ and MCA_v_, as well as the unknown insonation angle of the TCD probes (45).

### Data Exclusion

Of the 154 participants recruited to the study, 21 participants were classified as “circa PHV” (between −0.5 and 0.5 yr from PHV) and were excluded from the data analysis to address the study hypothesis. We were unable to insonate one of either the right MCA or left PCA concurrently in 12 participants during resting measures. Therefore, 121 participants were included in the analysis of resting cerebrovascular measures. Furthermore, 12 participants were unable to complete at least five cycles of the visual stimuli with all cerebrovascular and cardiorespiratory measures (because of either TCD headset/probe discomfort, mouthpiece discomfort, disturbance of finger photoplethysmography signal, or insufficient motivation to keep eyes closed between visual stimuli). A further three participants were excluded from the data analysis because of unacceptable PCA_v_ or MCA_v_ waveforms during the NVC assessment. Therefore, 106 participants were included in the final NVC analysis.

### Cardiorespiratory Fitness

Cardiorespiratory fitness was assessed via an incremental exercise test on an electronically braked cycle ergometer (Excalibur Sport, Lode, Gronigen, The Netherlands) to volitional exhaustion. Adjustments were made to the saddle and handlebars of the ergometer for each participant to ensure a comfortable cycling position. HR (RS400, Polar Electro, Kemple, Finland) and oxygen consumption (V̇o_2_) were assessed at rest and continuously throughout the exercise protocol (Oxycon Pro, Jaeger, Hoechberg, Germany). The exercise test used a ramp incremental protocol where workload increments were determined by participant stature and training status (46). Participants were encouraged to maintain a cadence of 75–85 rpm throughout the protocol. The test was ended once the participant failed to maintain a cadence ≥70 rpm for ≥5 consecutive seconds. Following 15 min of rest, participants completed a constant-load supramaximal verification test at 105% of power output achieved at peak V̇o_2_ during the incremental ramp test to confirm attainment of V̇o_2max_, as recommended for cardiorespiratory fitness testing in pediatric cohorts (28, 47). Individual V̇o_2max_ values were then allometrically scaled to lean body mass using a cohort determined exponent (LBM^0.93^) to account for developmental changes in lean body mass across adolescence (48).

### Statistical Analysis

Power analyses for data presented in this manuscript were conducted a priori by sampling pilot data assessing MCA_v_ via transcranial Doppler ultrasound in a similar cohort of pre (*n* = 12; 74.3 ± 7.1 cm·s^−1^)- and post (*n* = 12; 68.4 ± 5.8 cm·s^−1^)-PHV youth. The minimum required sample size for a statistically significant main effect of training status was *n* = 12 per group based on 90% power at a two-sided 0.05 significance level where f^2^ = 0.47. As such, we aimed to recruit at least 12 participants in each group to achieve statistical power for main effects of training status for baseline intracranial velocities, as well as allowing for data dropout during the NVC assessment. Statistical analysis was conducted on SPSS statistical software package (version 23.0, Chicago, IL). Normal distribution of outcome variables was confirmed via Shapiro–Wilk statistical tests and visual inspection of p-p plots. All data are presented as group means (SD) with statistical significance set to *P* < 0.05 unless otherwise stated. A three-factor analysis of variance (ANOVA) was used to determine the main effects of maturity status, biological sex, and training status, as well as the interaction effects of these variables on intracranial velocities and NVC. Post hoc comparisons with Bonferroni corrections were conducted to identify significant differences among groups when significant main or interaction effects were observed. The main aim of this study was to understand the influence of training status on intracranial velocities and NVC during different stages of maturity. As such, the reporting of post hoc comparisons will focus on the effect of training status on intracranial hemodynamics and NVC.

## RESULTS

### Descriptive Characteristics

Post-PHV youth had a higher maturity offset, chronological age, stature, body mass, LBM, and MAP than their pre-PHV counterparts (all; *P* ≤ 0.001, Table 1). In addition, post-PHV youth demonstrated greater training volumes compared with their pre-PHV counterparts (*P* ≤ 0.001), but V̇o_2max_ was similar in pre- and post-PHV youth (*P* = 0.580, Table 1). Endurance-trained youth had a higher training volume and V̇o_2max_ compared with untrained youth (both; *P* ≤ 0.001, Table 1).

### The Influence of Training Status on Baseline Intracranial Hemodynamics

**Figure 1.**
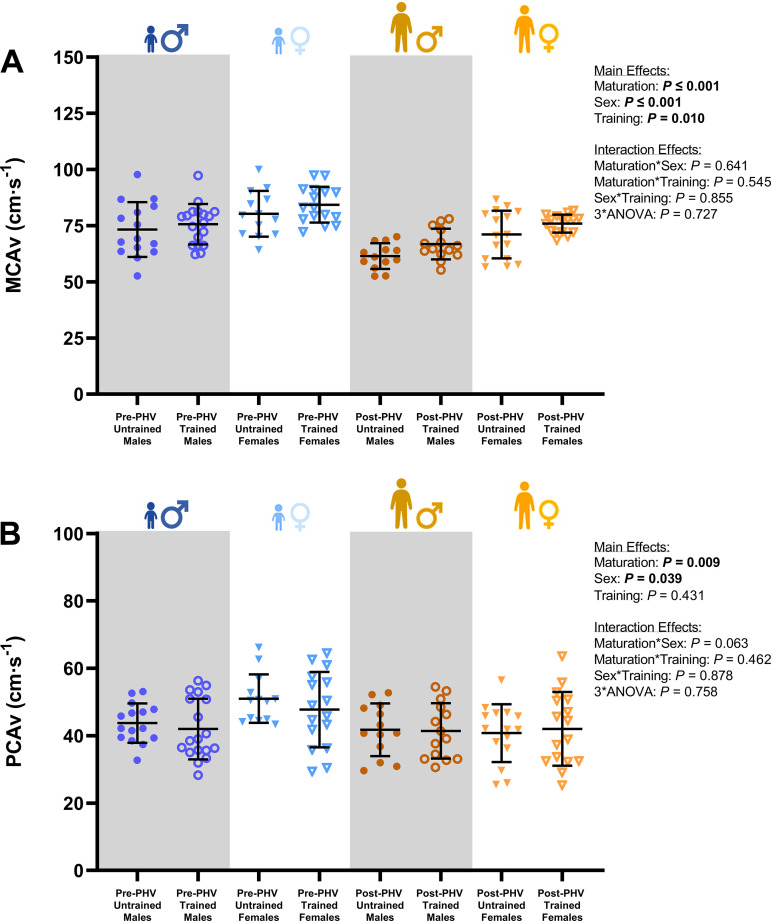
Group means for resting middle cerebral artery blood velocity (MCA_v_; *A*) and posterior cerebral artery blood velocity (PCA_v_; *B*) in males [pre-peak height velocity (PHV) untrained, *n* = 15; pre-PHV trained, *n* = 18; post-PHV untrained, *n* = 13; and post-PHV trained, *n* = 14] and females (pre-PHV untrained, *n* = 13; pre-PHV trained, *n* = 16; post-PHV untrained, *n* = 16; and post-PHV trained, *n* = 17) assessed via a three-way ANOVA. Error bars indicate group means (SD). *P* values outside of the figure plot indicate where significant main and interaction effects of maturity status, biological sex, and training status occurred. ANOVA, analysis of variance.

Endurance-trained youth demonstrated a higher resting MCA_v_ (*P* = 0.010) and MCA_CVC_ (*P* = 0.015) when compared with untrained counterparts (Table 2 and Fig. 1). However, post hoc comparisons revealed no differences in MCA_v_ and MCA_CVC_ between trained and untrained pre-PHV males (*P* = 0.443 and *P* = 0.198) and females (*P* = 0.217 and *P* = 0.186) or post-PHV males (*P* = 0.111 and *P* = 0.182) and females (*P* = 0.117 and *P* = 0.331). There was also a significant main effect for maturity status and biological sex on several metrics of resting cerebrovascular hemodynamics. Baseline MCA_v_ (*P* ≤ 0.001), PCA_v_ (*P* = 0.009), MCA_CVC_ (*P* ≤ 0.001), and PCA_CVC_ (*P* ≤ 0.001) were all lower in post-PHV youth when compared with pre-PHV counterparts (Table 1 and Fig. 1). Similarly, baseline MCA_v_ (*P* ≤ 0.001), PCA_v_ (*P* = 0.039), MCA_CVC_ (*P* ≤ 0.001), and PCA_CVC_ (*P* = 0.037) were lower in males when compared with females.

**Table 2.** Baseline cerebrovascular hemodynamics and cardiorespiratory variables across all groups

	Pre-PHV				Post-PHV				*P* Value (Interaction)						
	Untrained males	Trained males	Untrained females	Trained females	Untrained males	Trained males	Untrained females	Trained females	Maturation status	Sex	Training status	Maturation × sex	Maturation × training	Sex × training	Maturation × sex × training
*n*	15	18	13	16	13	14	16	17							
MCA_v_, cm·s^−1^	73.3 (12.2)	75.6 (9.0)	80.3 (10.1)	84.3 (8.0)	61.4 (5.7)	66.8 (6.8)	71.0 (10.6)	75.9 (5.0)	**≤0.001**	**≤0.001**	**0.010**	0.641	0.545	0.855	0.727
MCA_PI_, AU	0.74 (0.12)	0.78 (0.11)	0.75 (0.13)	0.82 (0.12)	0.89 (0.12)*	0.83 (0.17)	0.79 (0.08)†	0.74 (0.08)†	0.076	0.076	0.915	**0.006**	**0.011**	0.627	0.848
MCA_CVC_, cm·s·mmHg^−1^	0.97 (0.18)	1.02 (0.13)	1.06 (0.15)	1.13 (0.12)	0.76 (0.11)	0.83 (0.09)	0.91 (0.14)	0.96 (0.08)	**≤0.001**	**≤0.001**	**0.015**	0.385	0.905	0.862	0.767
PCA_v_, cm·s^−1^	38.6 (6.4)	41.9 (6.3)	50.4 (10.4)	45.7 (9.8)	37.4 (9.4)	35.7 (7.4)	40.2 (8.0)	42.4 (8.9)	0.105	**0.027**	0.178	0.556	0.233	0.389	0.778
PCA_PI_, AU	0.78 (0.15)	0.74 (0.09)	0.73 (0.15)	0.84 (0.15)†‡	0.84 (0.15)	0.89 (0.15)*	0.73 (0.07)†	0.77 (0.11)†	0.114	**0.049**	0.080	**0.004**	0.758	0.114	0.074
PCA_CVC_, cm·s·mmHg^−1^	0.59 (0.09)	0.57 (0.12)	0.68 (0.10)	0.64 (0.15)	0.52 (0.14)	0.52 (0.12)	0.54 (0.13)	0.53 (0.14)	**≤0.001**	**0.037**	0.438	0.159	0.639	0.820	0.858
RHR, beats/min	73 (9)	63 (7)‡	84 (12)	72 (9)	64 (9)	57 (7)	74 (6)	63 (8)	**≤0.001**	**≤0.001**	**≤0.001**	0.534	0.678	0.308	0.665
MAP, mmHg^−1^	76 (6)	74 (5)	76 (5)	75 (7)	81 (7)	81 (5)	78 (5)	80 (6)	**≤0.001**	0.333	0.845	0.239	0.319	0.347	0.920
$\text{P}{\text{ET}}_{{\text{CO}}_{2}}$, mmHg^−1^	38.9 (2.8)	40.2 (3.2)	38.6 (2.7)	39.8 (2.5)	41.2 (2.8)	41.0 (2.3)	39.7 (2.5)	41.2 (3.2)	0.089	0.422	0.570	0.797	0.562	0.523	0.102
$\text{P}{\text{ET}}_{{\text{O}}_{2}}$, mmHg^−1^	104.7	103.7	103.5	104.6	104.9	103.2	103.1	101.2	0.356	0.055	0.157	0.166	0.063	0.415	0.356

Values are means (SD); *n*, number of participants. MAP, mean arterial blood pressure; MCA_CVC_, middle cerebral artery conductance; MCA_PI_, middle cerebral artery pulsatility index; MCA_v_, middle cerebral artery blood velocity; PCA_CVC_, posterior cerebral artery conductance; PCA_PI_, posterior cerebral artery pulsatility index; PCA_v_, posterior cerebral artery blood velocity; PHV, peak height velocity; $\text{P}{\text{ET}}_{{\text{CO}}_{2}}$, end-tidal caron dioxide; $\text{P}{\text{ET}}_{{\text{O}}_{2}}$, end-tidal oxygen; RHR, resting heart rate. Group differences were assessed via three-way ANOVA. *Significant difference between pre- and post-PHV youth. †Significant difference between males and females. ‡Significant difference between trained and untrained youth. Boldface indicates significant values at *P* ≤ 0.05.

### The Influence of Training Status on Neurovascular Coupling

**Figure 2.**
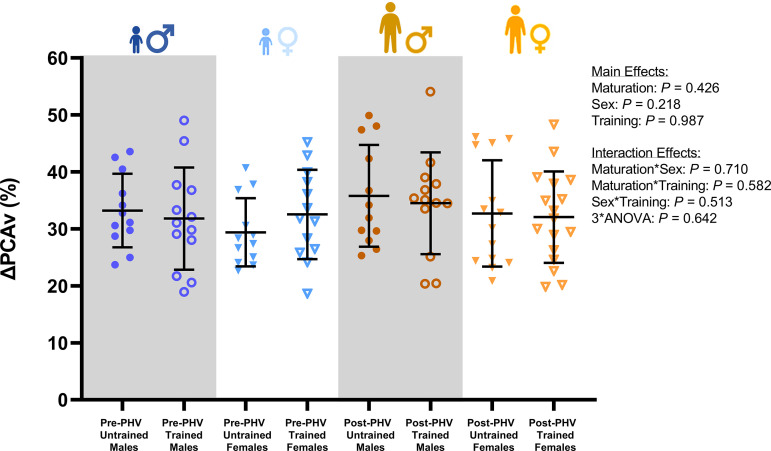
Group means for the percent change in posterior cerebral artery blood velocity (ΔPCA_v_) during the neurovascular coupling task in males [pre-peak height velocity (PHV) untrained, *n* = 12; pre-PHV trained, *n* = 13; post-PHV untrained, *n* = 12; and post-PHV trained, *n* = 13] and females (pre-PHV untrained, *n* = 12; pre-PHV trained, *n* = 13; post-PHV untrained, *n* = 14; and post-PHV trained, *n* = 17) assessed via a three-way ANOVA. Error bars indicate the group means (SD). *P* values outside of the figure plot indicate where significant main and interaction effects of maturity status, biological sex, and training status occurred. ANOVA, analysis of variance.

**Figure 3.**
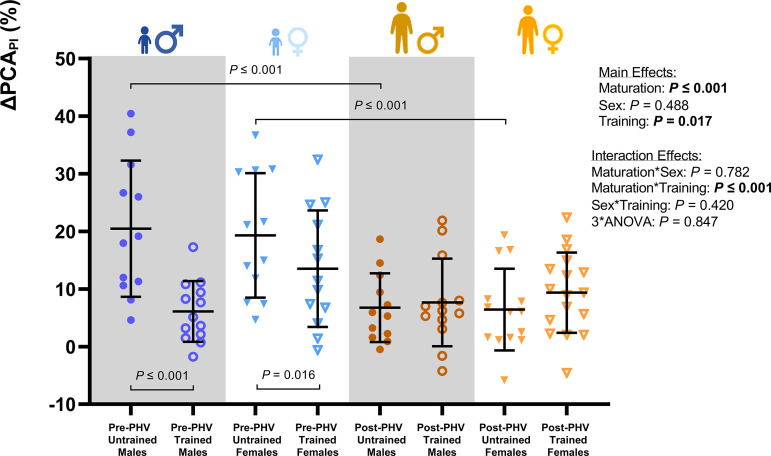
Group means for the percent change in posterior cerebral artery blood pulsatility index (ΔPCA_PI_) during the neurovascular coupling task in males [pre-peak height velocity (PHV) untrained, *n* = 12; pre-PHV trained, *n* = 13; post-PHV untrained, *n* = 12; and post-PHV trained, *n* = 13] and females (pre-PHV untrained, *n* = 12; pre-PHV trained, *n* = 13; post-PHV untrained, *n* = 14; and post-PHV trained, *n* = 17) assessed via a three-way ANOVA. Error bars indicate group means (SD). *P* values outside of the figure plot indicate where significant main and interaction effects of maturity status, biological sex, and training status occurred. *P* values within the figure plot indicate a significant difference between groups during post hoc comparisons. ANOVA, analysis of variance.

There was no effect of training status on the relative ΔHR [10.5 (9.2) vs. 12.2 (6.6) %; *P* = 0.312], ΔMAP [3.6 (2.6) vs. 3.5 (2.9) %; *P* = 0.789], or Δ$\text{P}{\text{ET}}_{{\text{CO}}_{2}}$ [10.7 (7.2) vs. 9.6 (7.0) %; *P* = 0.528] during the NVC assessment. Likewise, the relative ΔMCA_v_ and ΔPCA_v_ during the NVC assessment were not influenced by training status (*P* = 0.370 and *P* = 0.987; Table 3 and Fig. 2). The relative ΔMCA_PI_ during NVC was similar in trained and untrained youth during the NVC assessment (*P* = 0.717, Table 3), but the relative ΔPCA_PI_ was lower in trained youth when compared with untrained counterparts (*P* = 0.017, Table 3 and Fig. 3). Indeed, post hoc comparisons revealed that the relative ΔPCA_PI_ during the NVC assessment was lower in pre-PHV trained males (*P* ≤ 0.001) and females (*P* = 0.016) when compared with their untrained counterparts, but there were no training-related differences in post-PHV youth (*P* = 0.784 and *P* = 0.334). Furthermore, the relative ΔPCA_PI_ during the NVC assessment was lower in post-PHV untrained males and females compared with their pre-PHV counterparts (both; *P* ≤ 0.001), but there were no maturity-related differences in trained youth (*P* = 0.956 and *P* = 0.577). In addition, the relative ΔMCA_CVC_ during the NVC assessment was lower in trained youth compared with untrained youth (*P* = 0.037), whereas there was a significant maturity- and training-status interaction effect on the ΔPCA_CVC_ (*P* = 0.035). However, post hoc comparisons revealed no differences in the ΔMCA_CVC_ or ΔPCA_CVC_ between trained and untrained pre-PHV males (*P* = 0.442 and *P* = 0.197) and females (*P* = 0.490 and *P* = 0.152) or post-PHV males (*P* = 0.064 and *P* = 0.248) and females (*P* = 0.377 and *P* = 0.765) during the NVC assessment.

**Table 3.** Absolute and relative changes in cerebrovascular hemodynamics during neurovascular coupling task

	Pre-PHV				Post-PHV				*P* Value (Interaction)						
	Untrained males	Trained males	Untrained females	Trained females	Untrained males	Trained males	Untrained females	Trained females	Maturation status	Sex	Training status	Maturation × sex	Maturation × training	Sex × training	Maturation × sex × training
*n*	12	13	12	13	12	13	14	17							
ΔMCA_v_															
Absolute, cm·s^−1^	10.3 (5.9)	9.1 (7.3)	12.0 (7.0)	12.0 (6.7)	9.0 (3.1)	5.9 (3.2)	8.8 (4.2)	7.7 (4.8)	**0.012**	0.177	0.244	0.527	0.512	0.492	0.868
Relative, %	13.6 (6.8)	11.0 (6.0)	13.5 (5.1)	16.1 (7.8)	14.9 (7.1)	11.5 (8.2)	12.7 (4.6)	11.1 (5.6)	0.492	0.662	0.370	0.178	0.372	0.218	0.558
ΔMCA_PI_															
Absolute, AU	0.14 (0.11)	0.09 (0.08)	0.12 (0.15)	0.10 (0.07)	0.05 (0.05)*	0.07 (0.06)	0.07 (0.05)	0.11 (0.05)	**0.028**	0.375	0.832	0.357	**0.050**	0.429	0.933
Relative, %	19.8 (11.7)	15.3 (8.7)	15.0 (8.2)	13.3 (9.0)	7.8 (5.7)*	9.7 (7.1)	10.8 (9.4)	17.8 (8.0)†‡	**0.023**	0.556	0.717	**0.018**	**0.046**	0.287	0.768
ΔMCA_CVC_															
Absolute, cm·s·mmHg^−1^	0.13 (0.07)	0.13 (0.06)	0.18 (0.11)	0.18 (0.06)	0.15 (0.13)	0.09 (0.08)	0.14 (0.06)	0.12 (0.06)	0.109	0.138	0.271	0.288	0.395	0.574	0.760
Relative, %	14.9 (7.6)	12.1 (4.0)	19.0 (10.3)	16.8 (5.7)	19.5 (10.5)	13.1 (8.1)	16.0 (6.7)	13.3 (6.4)	0.886	0.406	**0.037**	0.074	0.541	0.524	0.650
ΔPCA_v_															
Absolute, cm·s^−1^	12.1 (4.9)	14.4 (5.7)	13.1 (4.0)	14.5 (4.4)	12.4 (2.4)	12.2 (4.2)	12.4 (5.2)	13.2 (4.5)	0.265	0.576	0.242	0.985	0.409	0.945	0.615
Relative, %	33.6 (8.1)	32.6 (9.5)	30.2 (8.0)	33.1 (9.3)	35.8 (8.9)	34.5 (8.9)	32.7 (9.3)	32.1 (8.0)	0.426	0.218	0.987	0.710	0.582	0.513	0.642
ΔPCA_PI_															
Absolute, AU	0.13 (0.13)	0.04 (0.04)	0.12 (0.11)	0.10 (0.08)	0.05 (0.06)	0.06 (0.07)	0.05 (0.04)	0.06 (0.05)	**0.003**	0.403	0.185	0.423	0.053	0.139	0.274
Relative, %	19.4 (13.5)	7.9 (6.0)‡	19.3 (10.8)	11.1 (7.3)‡	6.8 (6.0)*	7.7 (7.6)	6.4 (7.1)*	9.4 (7.0)	**≤0.001**	0.448	**0.017**	0.782	**≤0.001**	0.420	0.847
ΔPCA_CVC_															
Absolute, cm·s·mmHg^−1^	0.16 (0.07)	0.20 (0.06)	0.17 (0.06)	0.21 (0.08)	0.15 (0.05)	0.17 (0.08)	0,18 (0.07)	0.17 (0.06)	0.249	0.314	0.081	0.840	0.272	0.594	0.694
Relative, %	30.8 (8.8)	35.6 (7.5)	27.7 (8.7)	34.6 (8.2)	39.4 (12.0)*	35.8 (8.0)	35.0 (10.0)	34.0 (9.8)	**0.039**	0.157	0.503	0.628	**0.035**	0.597	0.703

Values are means (SD); *n*, number of participants. MCA_CVC_, middle cerebral artery conductance; MCA_PI_, middle cerebral artery pulsatility index; MCA_v_, middle cerebral artery blood velocity; PCA_CVC_, posterior cerebral artery conductance; PCA_PI_, posterior cerebral artery pulsatility index; PCA_v_, posterior cerebral artery blood velocity; PHV, peak height velocity. Group differences were assessed via three-way ANOVA. *Significant difference between pre- and post-PHV youth. †Significant difference between males and females. ‡Significant difference between trained and untrained youth. Boldface indicates significant values at *P* ≤ 0.05.

There was no effect of maturity status or biological sex on the peak relative ΔHR [12.1 (6.9) vs. 10.8 (8.8) %; *P* = 0.401 and 11.4 (9.7) vs. 11.4 (6.1) %; *P* = 0.886], ΔMAP [4.1 (3.0) vs. 3.1 (2.4) %; *P* = 0.055 and 3.8 (2.8) vs. 3.3 (2.7) %; *P* = 0.436], or Δ$\text{P}{\text{ET}}_{{\text{CO}}_{2}}$ [10.0 (7.1) vs. 10.1 (7.1) %; *P* = 0.970 and 9.8 (6.0) vs. 10.3 (7.9) %; *P* = 0.774] during the NVC assessment. Likewise, the peak relative ΔMCA_v_ and ΔPCA_v_ during the NVC assessment were not influenced by maturity status (*P* = 0.492 and *P* = 0.426) or biological sex (*P* = 0.662 and *P* = 0.218; Table 1 and Fig. 2). However, the relative ΔMCA_PI_ (*P* = 0.023) and ΔPCA_PI_ (*P* ≤ 0.001) were lower in post-PHV youth when compared with pre-PHV counterparts.

## DISCUSSION

The aim of this study was to investigate the impact of exercise training status on resting intracranial cerebrovascular hemodynamics and their response to a visual NVC task at different stages of maturity. For the first time, we report that *1*) endurance-trained youth demonstrate higher MCA_v_ but similar PCA_v_ to untrained counterparts, *2*) the magnitude of the relative ΔMCA_v_ and ΔPCA_v_ during a visual NVC task was similar in trained and untrained youth, and *3*) the ΔPCA_PI_ during NVC was lower in pre-PHV trained youths when compared with untrained counterparts. Collectively, our data indicate that exercise training elevates regional cerebral blood velocity during youth, but training-mediated adaptations in NVC are only attainable before the onset of somatic maturation, highlighting the importance of exercise training on cerebrovascular function throughout youth.

### Training-Status Modulates Middle Cerebral Artery Hemodynamics during Youth

Endurance-trained youth demonstrated elevated MCA_v_, but not PCA_v_, when compared with their untrained counterparts, independent of potential differences in cerebral perfusion pressure. Thus, it seems likely that exercise-mediated adaptions in cerebral perfusion (14) are particularly feasible in the anterior region of the adolescent brain and supported by a reduction in downstream cerebrovascular resistance that elevates intracranial blood velocities. Indeed, several anterior regions of the brain are associated with a protracted neural development in adolescent humans (49), which may provide a greater window of opportunity for their exposure to pubertal spikes in IGF-1 and brain-derived neutrophic factor for further exercise-mediated adaptations in neurogenesis (22, 50) and angiogenesis (14). Interestingly, resting MCA pulsatility was elevated in endurance-trained pre-PHV youth compared with untrained counterparts, but resting MCA pulsility was lower in endurance-trained youth post-PHV when compared with untrained counterparts. This observation indicates that a threshold of circulating IGF-1 achieved during puberty, which is not present during childhood, is needed to facilitate further exercise-mediated cerebral angiogenesis and reduce downstream cerebrovascular resistance. Thus, adolescents may experience structural remodeling of the cerebrovascular network in response to exercise-mediated pulsatile flow that subsequently dampens the pulsatility of cerebral perfusion. However, whether training-mediated adaptations in cerebrovascular hemodynamics result in remodeling of the larger upstream cerebrovascular arteries during childhood and adolescence requires further investigation.

We have also demonstrated that MCA_v_, but not PCA_v_, is lower in post-PHV youth when compared with their pre-PHV counterparts, whereas MCA_v_ was lower in males when compared with females, independent of changes in cerebral perfusion pressure. These findings support the assertion that cerebral perfusion, particularly in the anterior regions, declines across adolescence in a sex-specific manner, corresponding to structural and metabolic neural development across adolescence (5, 51). Furthermore, MCA pulsatility index, a metric of downstream cerebrovascular resistance, was lower in post-PHV females compared with males, but similar in pre-PHV males and females. Thus, cerebrovascular resistance at the level of the MCA appears to only diverge in males and females during adolescence despite sex-related differences in cerebral blood velocity that are present across childhood and adolescence. Accordingly, the divergence in pulsatility index in males and females during adolescence may be a consequence of vasoactive sex hormones (52) modulating basal cerebrovascular tone during adolescence without altering cerebral perfusion.

### Exercise-Trained Children Demonstrate Smaller Increases in Cerebrovascular Pulsatility during Neurovascular Coupling Assessments

Although the relative change in PCA_v_ and MCA_v_ was similar across groups during the NVC task, the change in PCA pulsatility was greater in pre-PHV untrained youths compared with their post-PHV untrained counterparts, as well as their pre-PHV-trained counterparts. Therefore, despite similar cerebral blood velocities and pulsatility at rest, alongside similar changes in cerebral perfusion during the visual stimulus, the pulsatility of blood flow during the NVC task is dampened by exercise training in pre-PHV youth. Indeed, evidence from adults indicates that the increase in PCA_v_ during NVC tasks is driven by the change in end-diastolic velocity rather than the peak systolic velocity (43). An increase in pulsatility occurs when there is a greater contribution of peak systolic velocity to the mean velocity of the waveform. Therefore, the peak systolic velocity has a greater contribution to the change in PCA_v_ during NVC tasks in children when compared with adolescents and adults. This transition from peak systolic- to end diastolic-mediated changes in PCA_v_ appears to be hastened by exercise training during childhood. We speculate that a higher resting cerebral perfusion and larger increases in cerebrovascular pulsatility during NVC in children relate to lower vascular tone at rest and an attenuated decline in cerebrovascular resistance during metabolic stimuli, when compared with adolescents and adults. This observation would highlight the transition from childhood to adolescence, or puberty, as a key period in the development of cerebrovascular function.

Given the evidence that cardiorespiratory fitness at 18 yr of age is associated with reduced incidence of neurocognitive disease later in life (7), it is likely that elevated cardiorespiratory fitness during adolescence promotes a healthy NVC phenotype across adulthood. However, it is important to consider that our cohort of trained adolescent youth had been exercise training for several years (i.e., since they were children) and had likely already experienced similar training-mediated adaptions in NVC as their younger counterparts. It is an intriguing possibility that functional adaptations in NVC during childhood are accompanied by complimentary structural adaptations in cerebrovascular remodeling or angiogenesis with continued endurance training into adolescence (14, 53) that make further functional changes in NVC at this early stage of life redundant. Indeed, cerebrovascular angiogenesis can be stimulated by exercise training in rodents (14) and humans (54). Moreover, exercised-mediated angiogenesis is dependent on the presence of IGF-1 in vivo (14), which is relatively low during childhood before peaking during adolescence (52). Therefore, exercise training during adolescence may result in further positive cerebrovascular adaptions that modulate aspects of NVC beyond the scope of this study. Regardless of the uncertainty of how exercised-mediated improvements in NVC translate from childhood to adolescence, it appears that exercise training has a positive impact on NVC during childhood. The exercised-mediated adaptations in NVC during childhood highlight the importance of physical activity for neurocognitive health during the formative developmental years, far earlier than the pathogenesis of neurocognitive disease. Accordingly, exercise training across childhood and adolescence may be vital for optimizing cerebrovascular function across the lifespan and delay the onset of neurocognitive disease.

There were no maturity or biological sex-related differences in the PCA_v_ response or the change in HR, MAP, $\text{P}{\text{ET}}_{{\text{CO}}_{2}}$, and $\text{P}{\text{ET}}_{{\text{O}}_{2}}$ during the NVC task. As such, our findings are not confounded by group differences in cerebral perfusion pressure or metabolic feedback mechanisms of cerebral blood flow regulation. The lack of maturity- or biological sex-related difference in the change in cerebral artery blood velocities during the NVC task conflicts with previous findings (10, 11), and is somewhat surprising given the contribution of NO-mediated vasodilation to NVC in adults (55) and the potential sex differences in the development of endothelial function across puberty (15, 26, 56). However, the conflicting findings are likely to be related to methodological differences in the NVC stimulus. Unlike previous reports (10, 11), the current study utilized a selective visual stimulus that is unlikely to be influenced by age- or learning-related differences across groups, while also invoking a large change in cerebral perfusion across all groups. Although it should be noted that the oxygen-carrying capacity of the blood increases across adolescence (2), the similar relative change in PCA_v_ in children and adolescents during the NVC task was present despite higher resting cerebral perfusion in children (5). Thus, our data show that children experience a larger absolute change in cerebral perfusion during the NVC task that counteracts the higher oxygen-carrying capacity in adolescent youth.

### Limitations

There are a few limitations that must be considered in the current study. First, $\text{P}{\text{a}}_{{\text{CO}}_{2}}$ was indirectly monitored via $\text{P}{\text{ET}}_{{\text{CO}}_{2}}$. However, $\text{P}{\text{ET}}_{{\text{CO}}_{2}}$ is commonly used as a suitable surrogate for $\text{P}{\text{a}}_{{\text{CO}}_{2}}$ during assessments of NVC (39, 40). Likewise, $\text{P}{\text{ET}}_{{\text{CO}}_{2}}$ has a strong relationship with $\text{P}{\text{a}}_{{\text{CO}}_{2}}$ in nonventilated children (57, 58), and the lack of change in $\text{P}{\text{ET}}_{{\text{CO}}_{2}}$ during the NVC task in the current study was similar across groups. Therefore, we are confident that our data provide novel insight into the influence of maturity and training status on NVC during youth. Second, we used PCA_v_ via TCD ultrasound to infer changes in cerebral perfusion during the NVC task. The larger proximal portions of the PCA (as insonated in the current study) appear to only dilate ≈0.9 ± 2.2% in response to similar visual stimuli (59). Therefore, we feel it is appropriate to use PCA_v_ as an index of cerebral perfusion during NVC stimuli because of the excellent temporal resolution of TCD (33) and the relatively small vasodilation of the P1 segment of the PCA. Finally, recent work has highlighted the important contribution of NO signaling to NVC in adults (55, 60). Likewise, endogenous sex hormones may play an important role in NO signaling and vascular function in adults (61, 62). We were unable to acquire suitable measurements of endothelial NO activity or sex hormones in the current study because of the ethical considerations associated with invasive venous blood sampling and pharmaceutical blockades in a nonclinical pediatric cohort.

### Conclusions

Our novel findings demonstrate that the change in cerebral perfusion during visual stimuli is unaffected by maturity or training status during youth. However, endurance-trained children demonstrated attenuated increases in cerebral blood flow pulsatility when compared with their untrained counterparts, whereas no training-mediated differences were present during the NVC task in adolescent youth. Therefore, exercise training during childhood, or before somatic maturation, modifies neurovascular coupling and may provide a foundation for additional cerebrovascular remodeling during adolescence that benefits long-term neurocognitive function.

## DATA AVAILABILITY

The data that support the findings of this study are available from the corresponding author upon reasonable request.

## GRANTS

This study was supported by The Waterloo Foundation (1129/4226; to M.S. and J.S.T.) and a FIFA Research Scholarship (to M.S. and J.S.T.).

## DISCLOSURES

No conflicts of interest, financial or otherwise, are declared by the authors.

## AUTHOR CONTRIBUTIONS

J.S.T., J.L.O., R.S.L., P.N.A., A.M.M., and M.S. conceived and designed research; J.S.T., D.R.P., T.G.D., A.J.M.D., T.D.G., C.T.R., K.O., and R.N.L. performed experiments; J.S.T. analyzed data; J.S.T., C.J.A.P., J.L.O., R.S.L., P.N.A., A.M.M., and M.S. .interpreted results of experiments; J.S.T. prepared figures; J.S.T. drafted manuscript; J.S.T., D.R.P., T.G.D., A.J.M..D., T.D.G., C.T.R., K.O., R.N.L., C.J.A.P., J.L.O., R.S.L., P.N.A., A.M.M., and M.S. edited and revised manuscript; J.S.T., D.R.P., T.G.D., A.J.M.D., T.D.G., C.T.R., K.O., R.N.L., C.J.A.P., J.L.O., R.S.L., P.N.A., A.M.M., and M.S. approved final version of manuscript.

## References

[1] Iadecola C. The neurovascular unit coming of age: a journey through neurovascular coupling in health and disease. Neuron 96: 17–42, 2017. doi:10.1016/j.neuron.2017.07.030. 28957666 PMC5657612

[2] Dallman PR, Siimes MA. Percentile curves for hemoglobin and red cellvolume in infnacy and childhood. J Pediatr 94: 26–31, 1979. doi:10.1016/s0022-3476(79)80344-3. 758417

[3] Huttenlocher PR. Synaptic density in human frontal cortex – developmental changes and effects of aging. Brain Res 163: 195–205, 1979. doi:10.1016/0006-8993(79)90349-4. 427544

[4] Knudsen EI. Sensitive periods in the development of the brain and behavior. J Cogn Neurosci 16: 1412–1425, 2004. doi:10.1162/0898929042304796. 15509387

[5] Satterthwaite TD, Shinohara RT, Wolf DH, Hopson RD, Elliott MA, Vandekar SN, Ruparel K, Calkins ME, Roalf DR, Gennatas ED, Jackson C, Erus G, Prabhakaran K, Davatzikos C, Detre JA, Hakonarson H, Gur RC, Gur RE. Impact of puberty on the evolution of cerebral perfusion during adolescence. Proc Natl Acad Sci USA 111: 8643–8648, 2014. doi:10.1073/pnas.1400178111. 24912164 PMC4060665

[6] Kwon D, Pfefferbaum A, Sullivan EV, Pohl KM. Regional growth trajectories of cortical myelination in adolescents and young adults: longitudinal validation and functional correlates. Brain Imaging Behav 14: 242–266, 2020. doi:10.1007/s11682-018-9980-3. 30406353 PMC6506406

[7] Nyberg J, Åberg MAI, Schiöler L, Nilsson M, Wallin A, Torén K, Kuhn HG. Cardiovascular and cognitive fitness at age 18 and risk of early-onset dementia. Brain 137: 1514–1523, 2014. doi:10.1093/brain/awu041. 24604561

[8] Schmithorst VJ, Badaly D, Beers SR, Lee VK, Weinberg J, Lo CW, Panigrahy A. Relationships between regional cerebral blood flow and neurocognitive outcomes in children and adolescents with congenital heart disease. Semin Thorac Cardiovasc Surg 10: 1285–1295, 2021. doi:10.1053/j.semtcvs.2021.10.014. 34767938 PMC9085965

[9] Xie C, Xiang S, Shen C, Peng X, Kang J, Li Y, Cheng W, He S, Banaschewski T, Barker GJ, Bokde ALW, Bromberg U, Büchel C, Desrivières S, Flor H, Grigis A, Garavan H, Gowland P, Heinz A, Ittermann B, Martinot J-L, Martinot M-LP, Nees F, Orfanos DP, Paus T, Poustka L, Fröhner JH, Smolka MN, Walter H, Whelan R; ZIB Consortium. A shared neural basis underlying psychiatric comorbidity. Nat Med 29: 1232–1242, 2023. doi:10.1038/s41591-023-02317-4. 37095248 PMC10202801

[10] Baller EB, Valcarcel AM, Adebimpe A, Alexander-Bloch A, Cui Z, Gur RC, Gur RE, Larsen BL, Linn KA, O'Donnell CM, Pines AR, Raznahan A, Roalf DR, Sydnor VJ, Tapera TM, Tisdall MD, Vandekar S, Xia CH, Detre JA, Shinohara RT, Satterthwaite TD. Developmental coupling of cerebral blood flow and fMRI fluctuations in youth. Cell Rep 38: 110576, 2022. doi:10.1016/j.celrep.2022.110576. 35354053 PMC9006592

[11] Schmithorst VJ, Vannest J, Lee G, Hernandez-Garcia L, Plante E, Rajagopal A, Holland SK; CMIND Authorship Consortium. Evidence that neurovascular coupling underlying the BOLD effect increases with age during childhood. Hum Brain Mapp 36: 1–15, 2015. doi:10.1002/hbm.22608. 25137219 PMC6869617

[12] Krause DN, Duckles SP, Pelligrino DA. Influence of sex steroid hormones on cerebrovascular function. J Appl Physiol (1985) 101: 1252–1261, 2006. doi:10.1152/japplphysiol.01095.2005. 16794020

[13] Cote S, Butler R, Michaud V, Lavallee E, Croteau E, Mendrek A, Lepage J-F, Whittingstall K. The regional effect of serum hormone levels on cerebral blood flow in healthy nonpregnant women. Hum Brain Mapp 42: 5677–5688, 2021. doi:10.1002/hbm.25646. 34480503 PMC8559491

[14] Lopez-Lopez C, LeRoith D, Torres-Aleman I. Insulin-like growth factor I is required for vessel remodeling in the adult brain. Proc Natl Acad Sci USA 101: 9833–9838, 2004. doi:10.1073/pnas.0400337101. 15210967 PMC470760

[15] Caulin-Glaser T, García-Cardeña G, Sarrel P, Sessa WC, Bender JR. 17 beta-estradiol regulation of human endothelial cell basal nitric oxide release, independent of cytosolic Ca^2+^ mobilization. Circ Res 81: 885–892, 1997. doi:10.1161/01.res.81.5.885. 9351464

[16] Bernini G, Versari D, Moretti A, Virdis A, Ghiadoni L, Bardini M, Taurino C, Canale D, Taddei S, Salvetti A. Vascular reactivity in congenital hypogonadal men before and after testosterone replacement therapy. J Clin Endocrinol Metab 91: 1691–1697, 2006. doi:10.1210/jc.2005-1398. 16492703

[17] Toth P, Tarantini S, Davila A, Valcarcel-Ares MN, Tucsek Z, Varamini B, Ballabh P, Sonntag WE, Baur JA, Csiszar A, Ungvari Z. Purinergic glio-endothelial coupling during neuronal activity: role of P2Y1 receptors and eNOS in functional hyperemia in the mouse somatosensory cortex. Am J Physiol Heart Circ Physiol 309: H1837–H1845, 2015. doi:10.1152/ajpheart.00463.2015. 26453330 PMC4698379

[18] Toth P, Tarantini S, Ashpole NM, Tucsek Z, Milne GL, Valcarcel-Ares NM, Menyhart A, Farkas E, Sonntag WE, Csiszar A, Ungvari Z. IGF‐1 deficiency impairs neurovascular coupling in mice: implications for cerebromicrovascular aging. Aging Cell 14: 1034–1044, 2015. doi:10.1111/acel.12372. 26172407 PMC4693458

[19] McAllister RM, Newcomer SC, Laughlin MH. Vascular nitric oxide: effects of exercise training in animals. Appl Physiol Nutr Metab 33: 173–178, 2008. doi:10.1139/H07-146. 18347669 PMC2646586

[20] Gielen S, Adams V, Linke A, Erbs S, Möbius-Winkler S, Schubert A, Schuler G, Hambrecht R. Exercise training in chronic heart failure: correlation between reduced local inflammation and improved oxidative capacity in the skeletal muscle. Eur J Cardiovasc Prev Rehabil 12: 393–400, 2005. doi:10.1097/01.hjr.0000174824.94892.43. 16079649

[21] Zaros PR, Pires CEMR, Bacci M, Moraes C, Zanesco A. Effect of 6-months of physical exercise on the nitrate/nitrite levels in hypertensive postmenopausal women. BMC Womens Health 9: 17, 2009. doi:10.1186/1472-6874-9-17. 19545388 PMC2714506

[22] Trejo JL, Carro E, Torres-Alemán I. Circulating insulin-like growth factor I mediates exercise-induced increases in the number of new neurons in the adult hippocampus. J Neurosci 21: 1628–1634, 2001. doi:10.1523/JNEUROSCI.21-05-01628.2001. 11222653 PMC6762955

[23] DHSC (Department of Health & Social Care). UK Chief Medical Officers’ Physical Activity Guidelines (Online). [Date accessed: 14 January 2023.] https://assets.publishing.service.gov.uk/government/uploads/system/uploads/attachment_data/file/832868/uk-chief-medical-officers-physical-activity-guidelines.pdf.

[24] Thomas KN, Lewis NCS, Hill BG, Ainslie PN. Technical recommendations for the use of carotid duplex ultrasound for the assessment of extracranial blood flow. Am J Physiol Regul Integr Comp Physiol 309: R707–R720, 2015. doi:10.1152/ajpregu.00211.2015. 26157060

[25] Hopkins ND, van den Munckhof I, Thijssen DHJ, Tinken TM, Cable NT, Stratton G, Green DJ. Are changes in conduit artery function associated with intima-medial thickness in young subjects? Eur J Prev Cardiol 20: 904–910, 2013. doi:10.1177/2047487312449294. 22584635

[26] Hopkins ND, Dengel DR, Stratton G, Kelly AS, Steinberger J, Zavala H, Marlatt K, Perry D, Naylor LH, Green DJ. Age and sex relationship with flow-mediated dilation in healthy children and adolescents. J Appl Physiol (1985) 119: 926–933, 2015. doi:10.1152/japplphysiol.01113.2014. 26251515 PMC4610003

[27] Port NL, Trimberger J, Hitzeman S, Redick B, Beckerman S. Micro and regular saccades across the lifespan during a visual search of ‘Where’s Waldo’ puzzles. Vision Res 118: 144–157, 2016. doi:10.1016/j.visres.2015.05.013. 26049037 PMC5851597

[28] Bhammar DM, Stickford JL, Bernhardt V, Babb TG. Verification of maximal oxygen uptake in obese and nonobese children. Med Sci Sports Exerc 49: 702–710, 2017. doi:10.1249/MSS.0000000000001170. 27875494 PMC5357186

[29] Baxter-Jones ADG, Eisenmann JC, Sherar LB. Controlling for maturation in pediatric exercise science. Pediatric Exerc Sci 17: 18–30, 2005. doi:10.1123/pes.17.1.18.

[30] Mirwald RL, Baxter-Jones ADG, Bailey DA, Beunen GP. An assessment of maturity from anthropometric measurements. Med Sci Sports Exerc 34: 689–694, 2002. doi:10.1249/00005768-200204000-00020. 11932580

[31] Silva DRP, Ribeiro AS, Pavão FH, Ronque ERV, Avelar A, Silva AM, Cyrino ES. Validity of the methods to assess body fat in children and adolescents using multi-compartment models as the reference method: a systematic review. Rev Assoc Med Bras (1992) 59: 475–486, 2013. doi:10.1016/j.ramb.2013.03.006. 24119380

[32] Slaughter MH, Lohman TG, Boileau RA, Horswill CA, Stillman RJ, Van Loan MD, Bemben DA. Skinfold equations for estimation of body fatness in children and youth. Hum Biol 60: 709–723, 1988. 3224965

[33] Willie CK, Cowan EC, Ainslie PN, Taylor CE, Smith KJ, Sin PYW, Tzeng YC. Neurovascular coupling and distribution of cerebral blood flow during exercise. J Neurosci Methods 198: 270–273, 2011. doi:10.1016/j.jneumeth.2011.03.017. 21459113

[34] Kim MO, Li Y, Wei F, Wang J, O'Rourke MF, Adji A, Avolio AP. Normal cerebral vascular pulsations in humans: changes with age and implications for microvascular disease. J Hypertens 35: 2245–2256, 2017. doi:10.1097/HJH.0000000000001459. 28692445

[35] Lu H, Xu F, Rodrigue KM, Kennedy KM, Cheng Y, Flicker B, Hebrank AC, Uh J, Park DC. Alterations in cerebral metabolic rate and blood supply across the adult lifespan. Cereb Cortex 21: 1426–1434, 2011. doi:10.1093/cercor/bhq224. 21051551 PMC3097991

[36] Purroy F, Sánchez E, Lecube A, Arqué G, Vicente-Pascual M, Mauri-Capdevila G, Torreguitart N, Hernández M, Barbé F, Fernández E, Pamplona R, Farràs C, Mauricio D, Bermúdez-López M, ILERVAS project. Prevalence and predictors of cerebral microangiopathy determined by pulsatility index in an asymptomatic population from the ILERVAS project. Front Neurol 12: 785640, 2021. doi:10.3389/fneur.2021.785640. 34970215 PMC8712482

[37] Janz KF, Dawson JD, Mahoney LT. Predicting heart growth during puberty: the muscatine study. Pediatrics 105: e63, 2000. doi:10.1542/peds.105.5.e63. 10799627

[38] Willie CK, Colino FL, Bailey DM, Tzeng YC, Binsted G, Jones LW, Haykowsky MJ, Bellapart J, Ogoh S, Smith KJ, Smirl JD, Day TA, Lucas SJ, Eller LK, Ainslie PN. Utility of transcranial Doppler ultrasound for the integrative assessment of cerebrovascular function. J Neurosci Methods 196: 221–237, 2011. doi:10.1016/j.jneumeth.2011.01.011. 21276818

[39] Caldwell HG, Coombs GB, Tymko MM, Nowak-Flück D, Ainslie PN. Severity-dependent influence of isocapnic hypoxia on reaction time is independent of neurovascular coupling. Physiol Behav 188: 262–269, 2018. doi:10.1016/j.physbeh.2018.02.035. 29458114

[40] Caldwell HG, Howe CA, Hoiland RL, Carr JMJR, Chalifoux CJ, Brown CV, Patrician A, Tremblay JC, Panerai RB, Robinson TG, Minhas JS, Ainslie PN. Alterations in arterial CO_2_ rather than pH affect the kinetics of neurovascular coupling in humans. J Physiol 599: 3663–3676, 2021. doi:10.1113/JP281615. 34107079

[41] Smirl JD, Wright AD, Bryk K, van Donkelaar P. Where’s Waldo? The utility of a complicated visual search paradigm for transcranial Doppler-based assessments of neurovascular coupling. J Neurosci Methods 270: 92–101, 2016. doi:10.1016/j.jneumeth.2016.06.007. 27291357

[42] Burma JS, Wassmuth RM, Kennedy CM, Miutz LN, Newel KT, Carere J, Smirl JD. Does task complexity impact the neurovascular coupling response similarly between males and females? Physiol Rep 9: e15020, 2021. doi:10.14814/phy2.15020. 34514743 PMC8436054

[43] Burma JS, Rattana S, Johnson NE, Smirl JD. Do mean values tell the full story? Cardiac cycle and biological sex comparisons in temporally derived neurovascular coupling metrics. J Appl Physiol (1985) 134: 426–443, 2023. doi:10.1152/japplphysiol.00170.2022. 36603050

[44] Phillips AA, Chan FH, Zheng MMZ, Krassioukov AV, Ainslie PN. Neurovascular coupling in humans: Physiology, methodological advances and clinical implications. J Cereb Blood Flow Metab 36: 647–664, 2016. doi:10.1177/0271678X15617954. 26661243 PMC4821024

[45] Gommer ED, Bogaarts G, Martens EGHJ, Mess WH, Reulen JPH. Visually evoked blood flow responses and interaction with dynamic cerebral autoregulation: correction for blood pressure variation. Med Eng Phys 36: 613–619, 2014. doi:10.1016/j.medengphy.2014.01.006. 24507691

[46] Perkins DR, Talbot JS, Lord RN, Dawkins TG, Baggish AL, Zaidi A, Uzun O, Mackintosh KA, McNarry MA, Cooper S-M, Lloyd RS, Oliver JL, Shave RE, Stembridge M. The influence of maturation on exercise-induced cardiac remodelling and haematological adaptation. J Physiol 600: 583–601, 2022. doi:10.1113/JP282282. 34935156

[47] Barker AR, Williams CA, Jones AM, Armstrong N. Establishing maximal oxygen uptake in young people during a ramp cycle test to exhaustion. Br J Sports Med 45: 498–503, 2011. doi:10.1136/bjsm.2009.063180. 19679577

[48] Loftin M, Sothern M, Abe T, Bonis M. Expression of VO_2_peak in children and youth, with special reference to allometric scaling. Sports Med 46: 1451–1460, 2016. doi:10.1007/s40279-016-0536-7. 27139725

[49] Casey BJ, Giedd JN, Thomas KM. Structural and functional brain development and its relation to cognitive development. Biol Psychol 54: 241–257, 2000. doi:10.1016/s0301-0511(00)00058-2. 11035225

[50] Aguiar AS, Speck AE, Prediger RDS, Kapczinski F, Pinho RA. Downhill training upregulates mice hippocampal and striatal brain-derived neurotrophic factor levels. J Neural Transm (Vienna) 115: 1251–1255, 2008. doi:10.1007/s00702-008-0071-2. 18629433

[51] Vandekar SN, Shou H, Satterthwaite TD, Shinohara RT, Merikangas AK, Roalf DR, Ruparel K, Rosen A, Gennatas ED, Elliott MA, Davatzikos C, Gur RC, Gur RE, Detre JA. Sex differences in estimated brain metabolism in relation to body growth through adolescence. J Cereb Blood Flow Metab 39: 524–535, 2019. doi:10.1177/0271678X17737692. 29072856 PMC6421255

[52] Cole TJ, Ahmed ML, Preece MA, Hindmarsh P, Dunger DB. The relationship between insulin‐like growth factor 1, sex steroids and timing of the pubertal growth spurt. Clin Endocrinol (Oxf) 82: 862–869, 2015. doi:10.1111/cen.12682. 25418044 PMC4949545

[53] Punglia RS, Lu M, Hsu J, Kuroki M, Tolentino MJ, Keough K, Levy AP, Levy NS, Goldberg MA, D’Amato RJ, Adamis AP. Regulation of vascular endothelial growth factor expression by insulin-like growth factor I. Diabetes 46: 8, 1997. doi:10.2337/diacare.46.10.1619. 9313759

[54] Weaver SR, Skinner BD, Furlong R, Lucas RAI, Cable NT, Rendeiro C, McGettrick HM, Lucas SJE. Cerebral hemodynamic and neurotrophic factor responses are dependent on the type of exercise. Front Physiol 11: 609935, 2020. doi:10.3389/fphys.2020.609935. 33551835 PMC7859714

[55] Hoiland RL, Caldwell HG, Howe CA, Nowak-Flück D, Stacey BS, Bailey DM, Paton JFR, Green DJ, Sekhon MS, Macleod DB, Ainslie PN. Nitric oxide is fundamental to neurovascular coupling in humans. J Physiol 598: 4927–4939, 2020. doi:10.1113/JP280162. 32785972

[56] Hutchison SJ, Sudhir K, Chou TM, Sievers RE, Zhu BQ, Sun YP, Deedwania PC, Glantz SA, Parmley WW, Chatterjee K. Testosterone worsens endothelial dysfunction associated with hypercholesterolemia and environmental tobacco smoke exposure in male rabbit aorta. J Am Coll Cardiol 29: 800–807, 1997. doi:10.1016/s0735-1097(96)00570-0. 9091527

[57] Berkenbosch JW, Lam J, Burd RS, Tobias JD. Noninvasive monitoring of carbon dioxide during mechanical ventilation in older children: end-tidal versus transcutaneous techniques. Anesth Analg 92: 1427–1431, 2001. doi:10.1097/00000539-200106000-00015. 11375819

[58] Nosovitch MA, Johnson JO, Tobias JD. Noninvasive intraoperative monitoring of carbon dioxide in children: endtidal versus transcutaneous techniques. Paediatr Anaesth 12: 48–52, 2002. doi:10.1046/j.1460-9592.2002.00766.x. 11849575

[59] Bizeau A, Gilbert G, Bernier M, Huynh MT, Bocti C, Descoteaux M, Whittingstall K. Stimulus-evoked changes in cerebral vessel diameter: a study in healthy humans. J Cereb Blood Flow Metab 38: 528–539, 2018. doi:10.1177/0271678X17701948. 28361587 PMC5851143

[60] O'Gallagher K, Rosentreter RE, Elaine Soriano J, Roomi A, Saleem S, Lam T, Roy R, Gordon GR, Raj SR, Chowienczyk PJ, Shah AM, Phillips AA. The effect of a neuronal nitric oxide synthase inhibitor on neurovascular regulation in humans. Circ Res 131: 952–961, 2022. doi:10.1161/CIRCRESAHA.122.321631. 36349758 PMC9770134

[61] Iwamoto E, Sakamoto R, Tsuchida W, Yamazaki K, Kamoda T, Neki T, Katayose M, Casey DP. Effects of menstrual cycle and menopause on internal carotid artery shear-mediated dilation in women. Am J Physiol Heart Circ Physiol 320: H679–H689, 2021. doi:10.1152/ajpheart.00810.2020. 33306444

[62] Orshal JM, Khalil RA. Gender, sex hormones, and vascular tone. Am J Physiol Regul Integr Comp Physiol 286: R233–R249, 2004. doi:10.1152/ajpregu.00338.2003. 14707008

